# Three-Dimensional Culture Systems in Gastric Cancer Research

**DOI:** 10.3390/cancers12102800

**Published:** 2020-09-29

**Authors:** George Alzeeb, Jean-Philippe Metges, Laurent Corcos, Catherine Le Jossic-Corcos

**Affiliations:** 1 Inserm, University Brest, EFS, UMR 1078, GGB, F-29200 Brest, France; george.alzeeb@univ-brest.fr (G.A.); laurent.corcos@univ-brest.fr (L.C.); 2 CHU de Brest, Inserm, University Brest, EFS, UMR 1078, GGB, F-29200 Brest, France; jean-philippe.metges@univ-brest.fr

**Keywords:** gastric cancer, spheroids, organoids, personalized medicine

## Abstract

**Simple Summary:**

It is getting more and more clear that cancer cell culture models are switching from two-dimension to three-dimensional, in order to better reflect in vivo situations where tumor cells have to cope with a highly interactive three-dimensional microenvironment. Several such culture models have been reported, predominantly multicellular tumor spheroids (MCTS) and patient-derived tumor organoids (PDTO). These are used both to investigate fundamental aspects of cancer development and as test systems for innovative therapies against gastric cancer, the fifth most common cancer and the third leading cause of cancer-related deaths worldwide. The authors review the actual state of research in this field to provide an overview of the contribution of MCTS and PDTO, especially in the areas of molecular profiling, drug discovery, pathogen infection, and personalized medicine.

**Abstract:**

Gastric cancer (GC), which includes cancer of the esophagus, the oesophagogastric junction, and the stomach fundus, is highly deadly with strong regional influence, Asia being the most affected. GC is often detected at late stages, with 30% of metastatic cases at diagnosis. Many authors have devised models to both unravel the mechanisms of GC development and to evaluate candidate therapeutics. Among these models, 2D-cell cultures are progressively replaced by 3D-cell cultures that recapitulate, much more comprehensively, tumor cellular and genetic heterogeneity, as well as responsiveness to environmental changes, such as exposure to drugs or irradiation. With respect to the specifics of GC, there are high hopes from such model systems, especially with the aim of identifying prognostic markers and novel drug targets.

## 1. Introduction

Gastric cancer (GC) is the fifth most common cancer and the third leading cause of cancer-related deaths worldwide, according to data from Global Cancer Statistics 2018 [1]. Adenocarcinomas of the esophagogastric junction (AEG) overlap histologically with GC and constitute an entity with rising incidence rates [2,3]. Lauren’s criteria are the most widely used to classify gastric adenocarcinomas, differentiating them histologically into intestinal and diffuse types [4]. Environmental factors such as *Helicobacter pylori* (*H. pylori*) infections, the greatest risk factor for GC [5], diet, and lifestyle are often associated with the intestinal type, while the diffuse type is more often associated with genetic abnormalities [6]. The Cancer Genome Atlas (TCGA) research network separated gastric adenocarcinomas into four different molecular subgroups: (i) positive for the Epstein–Barr virus (EBV) with frequent PIK3CA mutations and CDKN2A silencing, (ii) a microsatellite instable (MSI) subtype with a hyper-mutation phenotype, (iii) a genomically stable (GS) subtype displaying diffuse histology and frequent CDH1 and RHOA mutations, and iv) a chromosomal instable (CIN) subtype displaying aneuploidy and frequent mutations of TP53 as well as activation of the receptor tyrosine kinase (RTK)-RAS pathway [7]. The molecular characterization of AEG revealed their high similarity to the CIN subtype of GC [8]. The prognosis of GC is poor and most advanced forms of the disease still remain incurable [9]. Hence, GC treatment remains a major challenge and relies on surgical resection as the primary curative modality, i.e., for localized forms. Nonetheless, complementary approaches, such as neo-adjuvant and adjuvant chemotherapy (5-fluoruracil, oxaliplatin, docetaxel and epirubicin), have shown improved survival rates [10,11,12]. In addition, genetic alterations represent molecular targets for novel treatment options. So far, the only approved targeted therapies are: (i) anti-human epidermal growth factor receptor-2 monoclonal antibody “trastuzumab” and (ii) anti-vascular endothelial growth factor receptor-2 monoclonal antibody “ramucirumab” [13,14], while other therapeutic targets like Programmed cell Death protein 1 (PD-1) inhibition are under clinical investigation [15]. Although treatment effectiveness has improved during the past decade, GC survival rates remain poor [16]. This calls for an urgent need to develop innovative therapies available to GC patients. 

The conventional in vitro cancer model used to screen novel therapies is the monolayer two-dimension (2D) cancer cell line (CCL) culture [17]. 2D culture models impose unnatural geometric and mechanical constraints by adhering cells to an artificial substrate (plastic or glass). Such a culture affects cell polarity and therefore, potentially, tumor phenotypes. Three-dimensional (3D) culture systems have been developed to better mimic the functional aspects of tissues [18]. This results from (i) the organization of cells in layers with different proliferation rates, (ii) the formation of diffusion gradients of nutrients, oxygen and metabolic wastes, (iii) the specifics of cell-cell interactions, (iv) the expression of specific genes and (v) induction of chemoresistance [19,20,21]. Several 3D culture models have been reported [22], predominantly multicellular tumor spheroids (MCTS) [23] and patient-derived tumor organoids (PDTO) [24,25]. The main characteristics of these 3D models are presented in Table 1. 

MCTS models promote the formation of well-developed spheroids that resemble avascular tumor sites or micrometastatic regions in vivo [31]. Different co-culture approaches have been developed, including mixed populations of tumor cells and cancer associated fibroblasts (CAF), which showed increased ability to form spheroids [32]. Several studies validated the anticancer therapeutic potential of targeting the interactions between CAF and carcinoma cells [33]. However, using CCL to produce MCTS renders this approach hardly patient specific because most tumors contain highly heterogeneous subsets of cancer cells [34]. In vivo animal testing research is often employed for observing the effects on a living subject. The gold standard in vivo model for tumor development and analysis is patient-derived xenograft (PDX) [35]. Such a model conserves the clinical tumor architecture, the genotypic and phenotypic characteristics of the primary tumor as well as interactions with the microenvironment and the characteristics of patient’s tumor, recapitulating the inter-tumor heterogeneity [36]. However this still does not provide immediate clinically actionable data [37]. In addition, their use is quite expensive and time consuming, and raises ethical issues, at times where alternative in vitro/ex vivo models are gaining momentum. These reasons make in vivo models unsuitable for routine testing purposes and encourage the application of 3D cultures that permit recapitulating several mechanisms of drug resistance found in tumors in vivo.

Huge efforts in preclinical personalized therapy testing were explored by the recent development of PDTO as ex vivo models of human cancers, including GC [38]. Organoids are 3D cultures of multiple organ-specific cells of different types that can retain the morphologies and gene expression profiles of their organs of origin [39]. Organoids enable drug screening for personalized therapies as they provide genotypic stability and constitute a valuable tool to study pathogen infections [40,41]. A comparison of the main specificities of 2D, 3D, and PDX animal models is presented in Table 2.

In this review, we present the current status of in vitro / ex vivo 3D models of human GC as a surrogate to in vivo tumors. We describe established MCTS and PDTO methods in GC models and present an overview of important findings from different spheroids- and organoids-based studies, especially in the fields of molecular profiling, drug discovery, pathogen infection and personalized medicine. Lastly, we also attempt to propose ways for improving the relevance of next-generation 3D models.

## 2. Three D Multicellular Tumor Spheroid Model

**Figure 1:**
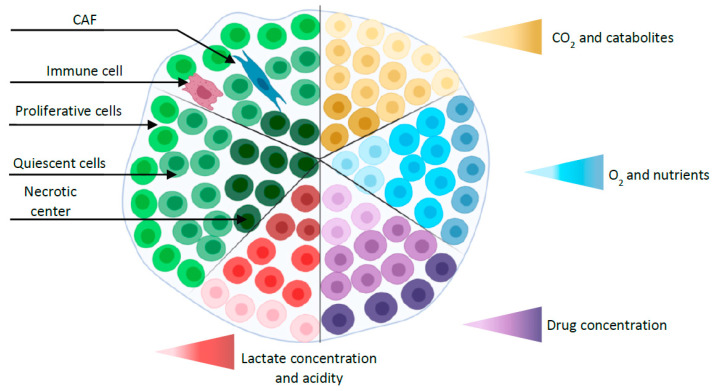
Simplified representation of a spheroid with different layers (proliferative outer layer, a quiescent intermediate layer, and the necrotic center) and gradients (oxygen, nutrients, CO_2_, catabolites, and drug concentration). Co-culture spheroid is represented by the addition of cancer-associated fibroblast (CAF) and immune cell.

Since Sutherland et al. established MCTS in the 1970s [46], this model has been one of the most commonly explored and characterized among the currently available 3D in vitro tumor models [47]. MCTS are aggregates of CCL grown with or without scaffolds representing avascular tumor nodules or micro-metastases [48]. Spheroids with diameters larger than 400–500 µm sustain oxygen and nutrient gradients associated with specific functional domains (proliferative outer layer, quiescent intermediate layer, and the necrotic center) [49] (Figure 1). As a result, protein and gene expression profiles of MCTS are closer to those of tumors than 2D tumor cell cultures [50]. In addition, MCTS can be constructed from tumor cells alone or combined to other cell types that can produce an extracellular matrix (ECM). They can be used to analyze the influence of 3D-specific cell-cell interactions on tumor progression, cell invasion or angiogenesis. As such, they are well suited to recapitulate the complexity and the cellular heterogeneity of tumors, a hallmark of cancer that may explain resistance to chemotherapy and participate in metastatic invasion. Hence, different 3D-co-culture approaches have been developed to analyze the interaction of tumor cells, fibroblasts, stem cells, adipocytes, or other cells present in the tumor microenvironment and to study the influence of these interactions on tumor progression or cell invasion [51,52,53].

### 2.1. MCTS Production Methodologies

Spheroid formation methodologies can be divided into two major categories: (i) scaffold-based models that take advantage of diverse natural (collagen, fibronectin, agarose, laminin, gelatin) [54], or synthetic (polyethylene oxide or polyethylene glycol) [55] materials to mimic in vivo tumor–ECM interactions [56] and (ii) scaffold-free models, which include mainly non-adherent, suspension, and hanging drop cultures, result in preventing cells attachment to the support, thereby forcing them to aggregate and form spheroids [49]. The plates used for this method are made non-adherent by coating them with an inert, non-cytotoxic and non-degradable substrate: agarose or poly-2-hydroxyethyl methacrylate (poly-HEMA) [57]. The principle of suspension culture methods is to keep the cells in suspension, either by agitation or by increasing medium viscosity (by addition of carboxy-methyl-cellulose) [58], but the spheroid size cannot be controlled, which can pose a problem when used in drug testing [59]. Finally, the hanging drop method involves cell suspension drops deposited on the underside of an adherent tissue culture lid. Cells aggregate at the bottom of the drop by gravity and form spheroids of uniform sizes [60]. However, the drops cannot exceed a volume of 50 µL in order to resist gravity [61]. The non-adherent surface method has been widely used for GC studies [62] (Table 3).

### 2.2. Applications of MCTS in Gastric Cancer

#### 2.2.1. Gene Expression Profiling

Genetic and epigenetic alterations contribute to the development and progression of multifactorial diseases such as GC [71]. Investigating the gene expression profiles of GC paves the way towards identifying novel diagnostic or prognostic biomarkers and developing future individualized medicine strategies. In vitro 3D experiments have gone a long way in understanding the molecular aspects of complex diseases [72]. CD44, a cell surface adhesion marker expressed by cancer stem cells (CSC) [73] has been reported as overexpressed in GC spheroids [64]. Oue et al. showed that KIFC1 and KIF11, two members of the kinesin-14 family, were overexpressed in spheroids compared to parental cells [74,75], while their knockdown inhibited spheroid formation [74,76]. In a similar context, this group also reported the under-expression of the claspin (CLSPN) gene, which codes for a nuclear protein involved in DNA replication and S-phase regulation, in spheroids [77]. Recently, Lee et al. demonstrated, using a limiting dilution protocol in a microwell-based culture chip, that gene expression of spheroid-forming cells was closely related to histological diffuse and intestinal type [78]. They observed an increase in expression levels of SOX2, a transcription factor expressed in stem cells), CD44 and E-cadherin in the diffuse type spheroid cell lines (SNU-638 and SNU-484) [79]. In addition, the expression of ERBB3 increased in spheroids made from intestinal type cell lines (MKN-28 and NCI-N87) [79]. miRNA expression was also investigated in GC MCTS models. Magalhães et al observed that the expression of has-miR-29c-5p, which regulated the expression of DNMT3A, CDC42, RCC2, and CDK6, was lower in the 3D model compared to 2D [26]. Changes in the microenvironment of the in vitro cells by 3D cultures can also impact on gene expression by modifying alternative splicing [80]. Indeed, a study by Branco da Cunha et al. showed an alternative splicing product of CD44 in GC spheroids, where the standard CD44 isoform (CD44s) was substituted by CD44 variant 6 (CD44v6) [81]. This increased progressively with the advancement of GC stages, from gastric pre-neoplastic lesions to advanced carcinoma [82]. Consequently, targeting the genes that distinguish MCTS from monolayer cell cultures introduces promising anticancer therapies. However, current studies on gene expression profiles of GC spheroids only scratch the surface and further studies need to be conducted to further clarify this process.

#### 2.2.2. Gastric Cancer Stem Cells: Biomarkers Identification

Cancer stem cells (CSC) are defined as a subpopulation of cancer cells that have a high capacity of self-renewal and differentiation into mature tumor cells, where multiple pathways are involved such as Notch, Wnt, Hedgehog and PI3K [83,84]. CSC constitute less than 5% of total tumor cells but they may play a crucial role as initiators of the heterogeneous lineage of cancer cells that constitute the tumor [85,86]. Because of their intrinsic resistance to anticancer drugs, CSC remains after chemotherapy or radiation therapy could be responsible for relapse after treatment. In addition, a poor prognosis of GC was associated with the expression of stem cell markers and related proteins, including CD44, SOX2 and OCT4/3 [87]. Nonetheless, gCSC markers have not been unambiguously identified [87]. For example, Rocco et al. reported that CD44^+^/CD133^+^ cells, detectable in primary GC, did not exhibit stem-like properties [88]. In this section, we will focus on studies that apply MCTS models to provide additional and better evidence of specific cell markers to identify gCSC. 

Takaishi et al. identified gCSC for the first time, using CD44 as a marker from a panel of human gastric CCLs. CD44^+^ cells could self-renew and form MCTS in a serum-free medium. CD44 knockdown reduced spheroid colony formation [89]. Han et al. reported that CD44^+^/EpCAM^+^ (Epithelial Cell Adhesion Molecule) cells grew exponentially in vitro as cancer spheres and had greater resistance to anticancer drugs than other subpopulations of cells. These results suggested that CD44^+^/EpCAM^+^ cells could be used as a model system for gCSC research [90], although these markers are not specific of gCSC. It resulted that spheroid body formation has been increasingly used as a functional approach for enriching in stem cell markers. Liu et al. were the first to develop spheroid body cells from human gastric CCL ‘MKN-45’. They demonstrated that these cells could generate greater numbers of new spheroid bodies than the parental cells and that spheroid body-forming cells were capable of self-renewal and proliferation, which are important CSC characteristics [91]. In addition, when cultured in stem cell conditioned media, these spheroid body-forming cells showed a significant overexpression of CD44 and ABCG2 (adenosine triphosphate binding cassette transporter G2) compared to the parental cells [92]. Furthermore, Zhang et al. found that spheroid cells from gastric CCL could self-renew and may also play roles in tumor initiation, chemo-resistance, and migration [87]. As already mentioned [79], using a limiting dilution protocol and a microwell-based culture chip to produce spheroids, Lee et al. demonstrated that these spheroids had larger populations of cells with stem cell-like properties, compared to spheroids formed by conventional tumor spheroid culture methods [78]. It is worth noting that these methods are hindered by poor single-cell seeding and low throughput. Other molecules have been reported as CSC-associated markers in GC. While Jiang et al. suggested that CD90 could be used to identify and isolate gCSC [93], Tian et al. documented a high expression of SOX2 in gastric MCTS and demonstrated the important role of SOX2 in sustaining stem cell properties [94]. In addition, using the MCTS method to isolate gCSC, Ptch and Gli1 (Sonic hedgehog (SHH) pathway target genes) were shown to be more expressed in MCTS cells than in adherent cells, suggesting that the SHH pathway was essential for the maintenance of CSC in human GC [95]. Ohkuma et al. demonstrated, using 3D invasion assays, that gastric CD71^−^ cell subpopulations had higher migratory and a more invasive potential compared to CD71^+^ cells, suggesting that low expression of CD71 could mark subpopulations of gCSC [96]. In addition, Yoon et al. found increased activity of RhoA in diffuse gCSC and a decreased spheroid formation after RhoA inhibition [97]. Despite this evidence, more studies are needed to further identify and characterize common gCSC biomarkers, especially as a means to better discriminate between CSC subpopulations, which will help to introduce better GC therapies [28,98].

#### 2.2.3. Drug Discovery

Standard 2D cell cultures have largely contributed to the development of many cancer therapies. However, the limitations of this model in reproducing in vivo tumor complexity and pathophysiology [99] may be one cause of the high attrition rate for cancer drugs entering early clinical trials [100]. Admittedly, culturing cells in 3D differentially impacts on their sensitivity to cytotoxic agents, as compared to 2D cultures, and usually makes them more resistant to treatment [101]. In this section, we will provide an overview of the implications of MCTS models in the development of anticancer drugs as well as in the discovery of novel treatment targets in GC. 

As discussed earlier in this review, gCSC are involved in tumor maintenance, resistance to treatments and tumor progression. Novel treatment modalities targeting gCSC have been developed using 3D models. Courtois et al. analyzed MCTS spheroid formation revealing CSC-presence and showed that metformin, an anti-diabetic drug with anti-proliferative effects, targeted gCSC, indicating that use of metformin could be a promising strategy to inhibit tumor growth [102]. Akrami et al. showed that ibuprofen, a nonsteroidal anti-inflammatory drug, prevented the initiation and the progression of GC [103]. They suggested that the anticancer effect of ibuprofen on gCSC was linked to inhibition of the Wnt/β-catenin signaling pathway [104]. After demonstrating its importance in sustaining CSC properties, Tian et al. proposed SOX2 as a potential target for GC therapy [94]. Similarly, Nishikawa et al. suggested that ALDH in gCSC may turn into a novel treatment target [105]. In addition, Koh et al. found that pantoprazole downregulated JAK2/STAT3 signaling, while inhibiting cellular migration or invasion in GC at the same time [106]. Because the efficacy of anticancer drugs relies on their ability to penetrate tumors efficiently, MCTS models are an ideal platform in view of their capacity to generate an ECM that obstructs drug penetration [107]. From that perspective, Sha et al. have constructed a recombinant protein named anti-EGFR-iRGD consisting of an anti-EGFR VHH (the variable domain from the heavy chain of the antibody to epidermal growth factor receptors) fused to iRGD, a tumor-specific binding peptide with high permeability. Anticancer activity and penetration of anti-EGFR-iRGD were evaluated with the MCTS model. Results from this study showed improvements in MCTS penetration as well as anti-GC efficacy when the anti-EGFR was fused with iRGD [27]. In addition, anti-EGFR-iRGD could enhance the efficacy of chemotherapeutic drugs, such as doxorubicin, bevacizumab, and placitaxel, in the inhibition of GC [27,108]. Furthermore, sTRAIL-iRGD, a recombinant protein with a high permeability index, displayed an anti-tumor effect in MCTS, with limited systemic toxicity [63,109]. Immunotherapy has had its fair share of applications using MCTS. Examples include exploring tumor immune escape mechanisms and screening immunotherapy agents or combinations pre-clinically. MCTS were also used for the evaluation of penetration and cytotoxicity of immune cells [110]. Zhou et al. established MCTS from a human gastric CCL to evaluate the cytotoxicity resulting from PD-1 blockade [111], a strategy to improve cancer therapy in the immuno-oncology field [112]. MCTS were formed in a medium containing IFN-γ and TNF-α to obtain PD-L1-expressing spheroids. The spheroids were then incubated with T cells in the absence or presence of PD-1 blockade. PD-1 blockade enhanced T-cell cytotoxicity against gastric spheroids expressing PD-L1 [111]. The potential of 3D culture models for the development of new anticancer strategies has evolved lately [113], demonstrating that CSC are more resistant to drugs than other malignant cells in the tumor aggregate [114]. Nonetheless, the heterogeneity of MCTS models could lead to reproducibility problems, leading to disputable biological information not well suited to test and select appropriate potential anticancer drugs [115].

## 3. Gastric Organoids

Although generic approaches, such as MCTS models, have participated in improving GC treatment, patient survival rates remain poor and there is still an urgent need to develop novel effective therapies with a model that would allow taking into account the genetic make-up of the individual tumor and provide immediate treatment selection. So-called organoids are one relevant option, although there is still no consensus on the definition of ‘organoid’ [39]. In general, organoids are in vitro 3D culture models grown from stem cells of primary tissues [116]. They can recapitulate key features and functions of their organs of origins such as architecture and gene expression profiles [117]. The many potential applications of this novel technology are beginning to be explored and used in many research areas, particularly in cancer research. The organoids co-culture approach can mimic the tumor immune microenvironment preserving T cell receptor and immune check point [118]. The first PDTO was established in 2011 when Sato et al. developed a long-term in vitro culture protocol for primary human epithelial cells isolated from small intestinal adenomas, metaplastic Barret epithelium and colon cancer tissues [119]. This innovation goes back to the identification of a particular intestinal stem cell marker, the leucine-rich repeat-containing G protein-coupled receptor 5 (Lgr5) by Barker et al. in 2007 [120]. Sato et al. next reported the first protocol that allowed establishing adult stem cells-derived organoids using Lgr5^+^ stem cells from intestinal crypts [121]. Since then, this protocol was applied to develop organoids from different organs including the pancreas [122], liver [123], esophagus [124], prostate [125], lung [126], breast [127], brain [116], and others [128]. Gastric organoids development was based on the localization of highly proliferative Lgr5^+^ gastric stem cells at the base of pyloric glands [129], shortly after the identification of Lgr5 as an intestinal stem cell marker [120]. This identification was facilitated by the fact that the gastric epithelium, like the intestinal epithelium, is constantly renewed and filled with rapidly proliferating stem cells. Stange et al. found, at the gland base of the gastric corpus, specialized chief cells marked by ‘Troy’. They demonstrated that a single Troy^+^ chief cell could generate gastric organoids [130]. In the following, we describe briefly the culture of patient-derived gastric cancer organoids (PDTO) and review important findings from organoids applications in GC studies.

**Figure 2:**
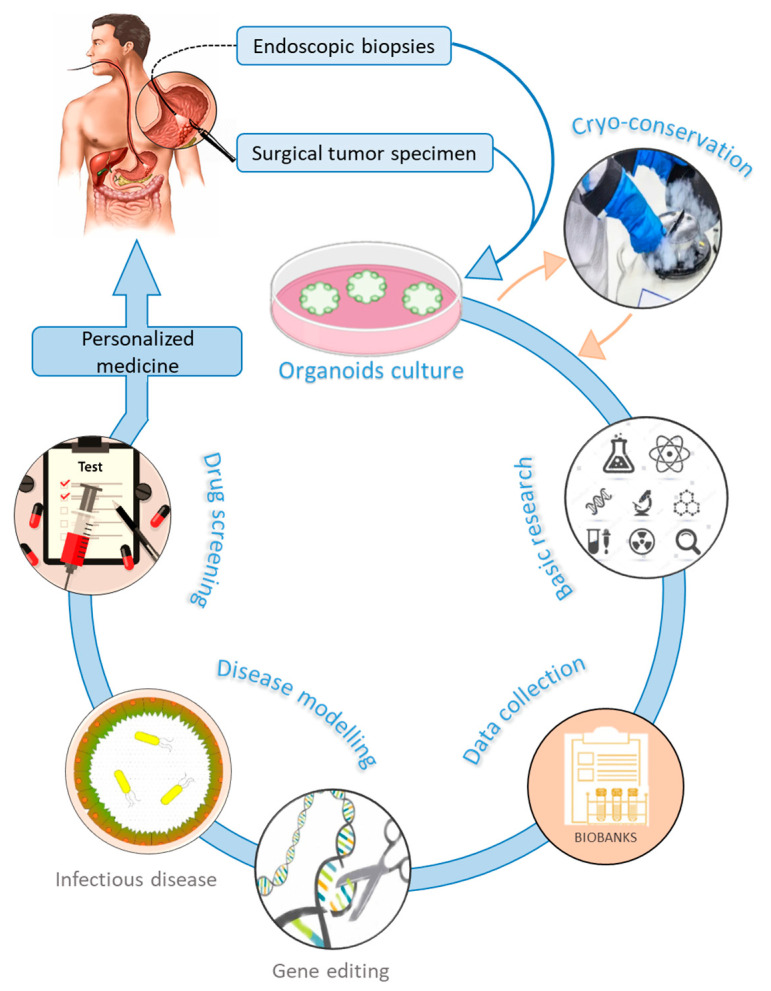
Graphical representation of gastric organoids applications and implication in personalized medicine.

Gastric organoids can be established from normal and cancerous gastric tissues. They are embedded into an ECM (matrigel) in a manner that recapitulates 3D in vivo conditions [129,131]. Methods used for culturing organoids from normal tissues have been adapted to successfully produce organoids from several human cancers [132]. PDTO can be propagated from surgical tumor specimens or endoscopic biopsies [133] (Figure 2). In general, protocols used to culture gastric organoids start from rinsing and mincing tumor tissues into small pieces (2–5 mm^3^). Released tumor cells from bulk tissues are then resuspended in matrigel [134] and overlaid with culture medium supplemented with essential components such as epidermal growth factor (EGF), noggin, R-spondin1, Wnt, fibroblast growth factor (FGF), gastrin, transforming growth factor (TGF), nicotinamide, insulin-like growth factor (IGF), and p38 inhibitor glycogen synthase kinase (GSK) [135]. These supplements make organoids culture environments very complex and different from conventional 2D culture media, which may limit strict comparisons in cell behavior. Gastric organoids tend to have a conserved architecture, with gastric glands budding around a central lumen [39]. However, contamination by epithelial and stromal cells, as well as the scarcity of cancer cells, represent a major challenge in culturing PDTO [130,136]. Primary PDTO appear like a mosaic of normal and cancer cells. Mechanical or enzymatic disruption allows passing organoids to maintain the culture for many months and even be cryo-preserved. In addition, PDTO biobanks were created either from primary [131,137] or metastatic tumors [138]. These biobanks offer a biological access to human GC, facilitate drug screening, validate biomarkers, and enable personalized medicine. However, statistical issues are raised, since too small a repertoire of banked cells might not be representative enough of the genetic heterogeneity of GC.

### 3.1. Applications of Organoids in Gastric Cancer

#### 3.1.1. Helicobacter Pylori Infection

*H. pylori* is a highly harmful human pathogen that infects approximately 60% of the world’s population [139]. *H. pylori* infection causes chronic gastritis, the major risk factor for GC development [140,141]. Injection of the cytotoxicity-associated gene A (CagA) by *H. pylori* into gastric epithelial cells induces pathogenesis [142]. Additionally, CagA upregulates Sonic Hedgehog (Shh), the regulator of gastric epithelial differentiation and function [143,144]. The majority of data generated on *H. pylori* pathogenesis was obtained from gastric CCL or in vivo animal models. However, the mechanisms of *H. pylori* infection that trigger GC initiation are poorly described. Bartfeld et al. developed a system to culture human gastric organoids from adult stem cells (aSCs) that can be productively infected by *H. pylori* [135]. Similarly, McCracken et al. reported the de novo generation of human gastric organoids through the direct differentiation of human pluripotent stem cells (hPSCs), to be used to model the pathophysiological response of the gastric epithelium to *H. pylori* [30]. Two hallmarks of *H. pylori* infection, the enhancement of gastric epithelial cells proliferation and the activation of nuclear factor-κB signaling, were discovered in 2D cell culture and were then validated in H. pylori-infected gastric organoids [135,145]. In addition, Wroblewski et al. showed that β-catenin was involved in the proliferation mechanism in H. pylori-infected organoids [146]. The activation of β-catenin and snail altered the expression and the localization of claudin-7, a protein implicated in the formation of tight junctions between epithelial cells [146]. In addition, Bertaux-Skeirik et al. have explored a new role of CD44 in *H. pylori*-induced proliferation, based on the fact that CD44 acts as a co-receptor for c-Met [147]. *H. pylori*-induced gastric pathology was contributed by the pathogen’s ability to colonize, alter and manipulate Lgr5^+^ progenitor-stem cells [148]. Moreover, Holokai et al. developed a human gastric organoid–immune cell co-culture system that allowed studying PD-L1 and PD-1 interactions, located on the gastric epithelial cells and the host’s immune cells, respectively, during *H.* pylori infection. They suggested that *H. pylori* infections modulate the PD-L1 immune checkpoint which may protect gastric epithelial cells against an immune response [149]. 

#### 3.1.2. Gastric Cancer Tumorigenesis

To date, the links between genotypes and phenotypes in the development of GC are poorly understood. Several transgenic animal models of GC tumorigenesis have been developed [150]. However, these models have all shown limitations linked to genetic background irrelevance, animal resistance and the inability to allow questioning the mechanisms that characterize the aggressive metastatic tumors. Recently however, organoids proved helpful for understanding the functional roles of driver gene mutations in the initiation and progression of cancers including colorectal [151] and gastric cancers. Knocking out CDH1, a tumor suppressor gene, Nanki et al. enhanced the transformation potential of normal gastric organoids to a diffuse GC morphology, indicating the implication of CDH1 in morphological and migratory features of GC. They showed occurrence of divergent genetic and epigenetic routes for developing WNT and R-spondin niche independency. In addition, they suggested that the loss of CDH1 and TP53 induced R-spondin independency uniquely during gastric tumorigenesis [137]. Another study by Sethi et al. showed that knocking out both CDKN2A and TP53 in dysplastic gastric organoids promoted cancer phenotypes [29]. Chen et al. investigated the role of epithelium–stroma interaction in the progression and the maintenance of gastric organoids. They demonstrated that Trp53^−/−^ macrophages present in the early stroma affected wound healing and tumor promotion. Additionally, they identified R-spondin 3 as an endogenous source supplied by fibroblasts that could sustain the growth niche in gastric tissue homeostasis and early cancer development [152]. Wang et al. demonstrated that silencing C8orf76 (chromosome 8 open reading frame 76), a booster of GC cell proliferation, suppressed tumor growth in PDTO [153]. Hence, the organoid model proved highly pertinent to identify several human molecular pathways associated with disease progression.

#### 3.1.3. Drug Sensitivity and Personalized Medicine

Intratumor heterogeneity [154] accounts for a large part of the limited benefits of current treatments. PDTO is a powerful ex vivo tool to take into account the genetic heterogeneity of primary tumors [155]. Drug exposure of organoids established from tumors obtained from seven patients treated with epirubicin, oxaliplatin, and 5-fluorouracil was correlated with the response of the primary tumor in each patient [156]. In a similar study, Li et al. demonstrated that malignant ascites-derived organoids preserved the histological architecture and the genomic landscape of the corresponding malignant ascite tumor cells, a common manifestation in advanced GC [157]. Vlachogiannis et al. showed the clinical potential of PDTO for selecting the best treatment option in cancers using a compound library of drugs. They also showed their capacity to recapitulate patient responses. Treatment with lapatinib, a tyrosine kinase inhibitor that targets the EGFR and HER2 tyrosine kinases, was effective against ErbB2-amplified PDTO compared to wild-type PDTO [138]. The evolution of translational research, through its applications with PDTO models, makes it emerge as a crucial strategy in personalized medicine programs [131,158]. New clinical trials are required to further validate the benefits of GC PDTO in personalized medicine, i.e., assessing the correlation between the in vivo primary tumor response and the ex vivo drug-mediated cytotoxicity. The OPPOSITE trial [159] is aimed at filling this gap.

## 4. Concluding Remarks

In this article, we attempted to provide an overview of the development of the major 3D cell culture models of human GC. This rapidly evolving field, which comprises mainly spheroid and organoid structures, aims at providing an ex-vivo alternative to the quite demanding and expensive PDX in vivo system. Hence, MCTS systems are well suited to analyze the interactions between the cells that compose the tumor, including CSC, CAF, immune and endothelial cells. As such, they are also convenient to analyze the effects of cytotoxic drugs, as well as to identify novel biomarkers. Alternatively, organoids have proven quite useful to address issues such as the contribution of PD-L1/PD1 from immune cells to the susceptibility to infection by helicobacter pylori or the specific roles of genes and gene pathways in gastric tumorigenesis and the response of cancer cells to chemotherapeutic drugs. Hence, these ex-vivo cell culture systems already represent plausible alternatives to PDX or to other animal models. Still, harmonization of techniques is needed to ensure better data reproducibility from the use of 3D models, before these can be seen as the gold standard for the preclinical screening of therapeutic strategies for GC.

## Figures and Tables

**Table 1:** Comparison between spheroids and organoids. (++ favorable, + possible), See references [26,27,28,29,30] for examples of studies that used either spheroids or organoids methodologies.

3D Culture Models	Spheroids	Organoids
Origin	Cancer cell lines	Patient tumor
Protocol	Easy to use	Delicate
Architecture	Simplified	Closer to organ
Reproducibility	High	Medium-high
Cellularity	Defined cell type	Different cell types
Cancer subtype modeling	+	++
Biological material produced	Abundant	Abundant
Genetic manipulation	++	++
Co-culture	++	+
High-throughput drug screening	++	+
Prediction of clinical drug response in vitro	+	++
Cost	Low	Medium
Biobanks	Unavailable	Available

**Table 2:** Comparison between 2D, 3D cell culture and PDX animal models [42,43,44,45].

Test System Main Features	2D Cell Culture [42,43]	3D Cell Culture [44]	PDX Animal Model [45]
Physiological relevance	Limited	Better than 2D standard culture	Most physiological environment
Model complexity	Limited	Complex	Very complex
Gene expression	Stable at early passages	Close to in vivo tumors	Close to in vivo tumors
Immune system	No but co-cultures of cancer cells and immune cells possible	No but co-cultures of cancer cells and immune cells possible	No
Efficient drug screening	Yes	Yes	No
Disease modeling	Naive	Feasible	Sophisticated methods
Data provider	Easily exploitable	Easily exploitable	Hardly exploitable
Controlled microenvironment	Yes	Yes	No
Reproducibility	Favorable	Not so favorable	Unsuited
Cost	Low	Some expensive materials and special equipment required	High
Ethical and regulatory issues	No	No	No

**Table 3:** Description, advantages and disadvantages of commonly used MCTS culture techniques. (* Refers to studies on colorectal cancer).

Spheroids Production Methods	Description	Advantages	Disadvantages	References
Scaffold-based models	3D construction that provides an ECM capable of supporting cells	Simple Mimic in vivo microenvironment Cell-ECM interactions Long-term culture Directly visualize and analyze	Difficulty of cell recovery Scaffold consistency can be variable across lots Nonuniform control (composition, size) Co-culture delicate Not suitable for drug testing	[27,63]
Non-adherent surfaces	Prevent attachment to the support	Simple Available pre-coated plates Uniform spheroid size control Ease of cell recovery Long-term culture Co-culture feasible Useful for drug screening Directly visualize and analyze	Low throughput production Defined co-culture cellular ratio Requires transfer of spheroids for analysis	[64,65,66,67]
Suspension culture	Keeps the cells in suspension to avoid sedimentation	High throughput production Homogeneous media composition Long-term culture	High shear force Nonuniform control (composition, size) Not easily suitable for drug testing Requires special equipment Requires a centrifugation step	[23] *
Hanging drop technique	Gravity based spheroid formation	Simple Uniform spheroid size control Co-culture feasible	Small size of spheroids Low throughput production Tedious spheroid handling and transfer Difficulty of long-term culture Defined co-culture cellular ratio Not suitable for drug testing	[68,69,70] *
